# Resting State fMRI Reveals Diminished Functional Connectivity in a Mouse Model of Amyloidosis

**DOI:** 10.1371/journal.pone.0084241

**Published:** 2013-12-17

**Authors:** Disha Shah, Elisabeth Jonckers, Jelle Praet, Greetje Vanhoutte, Rafael Delgado y Palacios, Christian Bigot, Dany V. D’Souza, Marleen Verhoye, Annemie Van der Linden

**Affiliations:** 1 Bio-Imaging Laboratory, University of Antwerp, Antwerp, Belgium; 2 F. Hoffmann-La Roche Pharmaceuticals Ltd, Neuroscience Discovery, Basel, Switzerland; Max Planck Institute for Human Cognitive and Brain Sciences, Germany

## Abstract

**Introduction:**

Functional connectivity (FC) studies have gained immense popularity in the evaluation of several neurological disorders, such as Alzheimer’s disease (AD). AD is a complex disorder, characterised by several pathological features. The problem with FC studies in patients is that it is not straightforward to focus on a specific aspect of pathology. In the current study, resting state functional magnetic resonance imaging (rsfMRI) is applied in a mouse model of amyloidosis to assess the effects of amyloid pathology on FC in the mouse brain.

**Methods:**

Nine APP/PS1 transgenic and nine wild-type mice (average age 18.9 months) were imaged on a 7T MRI system. The mice were anesthetized with medetomidine and rsfMRI data were acquired using a gradient echo EPI sequence. The data were analysed using a whole brain seed correlation analysis and interhemispheric FC was evaluated using a pairwise seed analysis. Qualitative histological analyses were performed to assess amyloid pathology, inflammation and synaptic deficits.

**Results:**

The whole brain seed analysis revealed an overall decrease in FC in the brains of transgenic mice compared to wild-type mice. The results showed that interhemispheric FC was relatively preserved in the motor cortex of the transgenic mice, but decreased in the somatosensory cortex and the hippocampus when compared to the wild-type mice. The pairwise seed analysis confirmed these results. Histological analyses confirmed the presence of amyloid pathology, inflammation and synaptic deficits in the transgenic mice.

**Conclusions:**

In the current study, rsfMRI demonstrated decreased FC in APP/PS1 transgenic mice compared to wild-type mice in several brain regions. The APP/PS1 transgenic mice had advanced amyloid pathology across the brain, as well as inflammation and synaptic deficits surrounding the amyloid plaques. Future studies should longitudinally evaluate APP/PS1 transgenic mice and correlate the rsfMRI findings to specific stages of amyloid pathology.

## Introduction

Alzheimer’s disease (AD) is a neurodegenerative disorder, that is characterised by memory loss and other cognitive impairments that eventually impede activities of daily life. AD pathology includes, among other things, the accumulation of amyloid, the formation of tau-fibrils, neuron loss and neuroinflammation [1]. AD is a complex disease, the cause of which is still unknown, and has been the subject of extensive research. 

Among AD-based research, non-invasive imaging studies that use techniques such as magnetic resonance imaging (MRI) and positron emission tomography [2] have a significant role. MRI studies [2] of AD include structural assessments of brain atrophy, hippocampal volume changes and ventricle enlargement. In addition to identifying structural changes, MRI also provides the possibility of studying functional alterations by means of functional MRI (fMRI). Functional changes occur much earlier than structural alterations [3], and there is therefore a high level of interest in these studies. A field that has gained immensely in popularity over recent years is resting-state fMRI (rsfMRI), which is used to study functional connectivity (FC) in the brain. 

FC is defined as the temporal correlation between low-frequency fluctuations in the blood-oxygen-level-dependent (BOLD) fMRI signal in distinct brain areas. The very first rsfMRI study, performed by Biswal et al., showed that motor regions activated after a motor task had highly correlated low-frequency fluctuations at rest, indicating that correlation of low-frequency fluctuations is a manifestation of brain FC [4,5]. Several rsfMRI studies have been performed in patients, assessing FC in a plethora of human diseases including epilepsy [6], multiple sclerosis (MS) [7] and AD [8]. RsfMRI studies have also been performed in monkeys [9] and rodents [10–12], demonstrating the existence of functional networks in the non-human brain.

In AD patients, rsfMRI has demonstrated altered FC in brain regions that are involved in memory functions, such as the hippocampus and the posterior cingulate cortex [8]. However, it is not known what causes these FC changes. The AD patients studied exhibited various aspect of AD pathology, including amyloid plaque formation, tau-pathology, and neuron loss, rendering it hard to correlate the FC findings to a specific aspect of disease. In mouse models of AD, however, it is much easier to make correlations as the models mimic only certain aspects of AD, and due to their short life span they can be followed up longitudinally. The first FC study in an AD mouse model was performed by Bero and colleagues using functional connectivity optical intrinsic signal (fcOIS) imaging [13] and demonstrated an age dependent decline of cortical interhemispheric FC in AD mice, that was associated with amyloid beta (Aβ) deposition. FcOIS imaging, however, is limited in its ability to assess regions localised deep in the brain, such as the thalamus and the hippocampus [14], the latter of which is a particularly relevant brain region in AD. 

RsfMRI could be an appropriate way to evaluate FC in mice, as it can be used to study cortical as well as subcortical regions, is non-invasive, and has high spatial and temporal resolution. RsfMRI has been performed in healthy mice for the first time at our laboratory, and we used independent component analysis (ICA) to demonstrate the presence of relevant functional connections in the mouse brain [11]. The possibility of performing rsfMRI in mouse models of disease opens up a whole new array of neurological mechanistic studies that can be performed. One example is the use of rsfMRI to study the effect of cage enrichment in a mouse model of AD [15].

Amyloidosis is one of the main pathological events in AD and, according to the amyloid cascade hypothesis, it can even be considered as the disease's lead contributing factor [16]. It is known that soluble amyloid, which eventually aggregates to form plaques, exerts toxic effects at the level of the synapses [17]. Altered FC in the brain has been demonstrated in human subjects harbouring amyloid plaques but no AD symptoms [18]. However, as mentioned before, it is not straightforward to correlate rsfMRI findings to specific pathological alterations in humans so it is unclear what exactly causes this altered FC. In this study, we used rsfMRI to assess FC in a mouse model of amyloidosis at an age when amyloid pathology is extensively present in the entire brain, without the occurrence of tau-pathology or neuron loss. We hypothesised that FC in most brain regions would differ between transgenic (TG) mice and age-matched wild-type (WT) mice. This hypothesis was supported by our results, which showed an overall decrease in FC in the brains of TG mice compared to WT mice.

## Materials and Methods

### Ethics statement

All procedures were performed in strict accordance with the European guidelines for the care and use of laboratory animals (86/609/EEC). The protocols were approved by the Committee on Animal Care and Use at the University of Antwerp, Belgium (permit number 2012-06) and all efforts were made to minimize animal suffering.

### Animals

The mouse model that was used in this study is the APP_KM670/671NL_PS1_L166P_ model [19,20]. This is a model of amyloidosis and mice show amyloid plaque deposition in the neocortex starting from approximately 2 months of age. Altogether, 18 male C57BL/6 mice of (18.9 ± 1.3) months old were subjected to MR imaging i.e. 9 wild-type (WT) animals and 9 APP_KM670/671NL_PS1_L166P_ transgenic (TG) animals. For the handling procedures the mice were anaesthetised with 2% isoflurane (IsoFlo, Abbott, Illinois, USA), which was administered in a mixture of 70% N2 and 30% O2. During the imaging procedure, medetomidine (Domitor, Pfizer, Karlsruhe, Germany) was administered first as a bolus injection of 0.3 mg/kg, then by a continuous infusion of 0.6 mg/kg/h, as was performed in a previous rsfMRI study at our laboratory [11]. After the imaging procedures, the effects of medetomidine were counteracted by the injection of 0.1mg/kg atipamezole (Antisedan, Pfizer, Karlsruhe, Germany). The physiological status of all animals was monitored throughout the imaging procedure. A pressure sensitive pad (MR-compatible Small Animal Monitoring and Gating system, SA Instruments, Inc.) was used to monitor breathing rate and a rectal thermistor with feedback controlled warm air circuitry (MR-compatible Small Animal Heating System, SA Instruments, Inc.) was used to maintain body temperature at (37.0 ± 0.5)°C 

### MRI

MRI procedures were performed on a 7T Pharmascan system (Bruker BioSpin, Germany). Images were acquired using a Bruker cross coil set-up with a transmit quadrature volume coil and a receive-only surface array for mice. Three orthogonal multi-slice Turbo RARE T2-weighted images were acquired to render slice-positioning uniform (repetition time 2000 ms, echo time 15 ms, 15 slices of 0.4 mm). Field maps were acquired for each animal to assess field homogeneity, followed by local shimming, which corrects for the measured inhomogeneity in a rectangular VOI within the brain. Resting-state signals were measured by a T2*-weighted single shot echo planar imaging (EPI) sequence with repetition time 2000 ms and echo time 15 ms. Fifteen axial slices of 0.4 mm with a gap of 0.1 mm were acquired with a bandwidth of 400 kHz, a field-of-view of (20x20)mm^2^ and a matrix size of (128 x 128), resulting in voxel dimensions of (0.156 x0.156)mm^2^. A total of 150 EPI datasets were acquired per animal, resulting in a scanning time of 5 minutes.

### Pre-processing

Pre-processing of the data, including realignment, a normalisation step, and smoothing, was performed using SPM8 software (Statistical Parametric Mapping, http://www.fil.ion.ucl.ac.uk) as previously described by Jonckers and colleagues [11]. Cerebral brain volume was calculated for each animal using the anatomical multi-slice T2-weighted images and AMIRA software (Amira, Template Graphics Software, Inc., San Diego, California, USA) and no significant differences were observed in the cerebral brain volumes of the WT mice (304 ±10)mm^3^ and TG mice (311±9)mm^3^. All rsfMRI data were normalised to the EPI image of a control animal. The normalisation steps consist of a global 12-parameter affine transformation followed by the estimation of the nonlinear deformations. Smoothing was performed using a Gaussian kernel of (0.3x0.3) mm^2^. 

### RsfMRI Analysis

FC between two brain regions is computed by calculating the correlation of the low-frequency fluctuations of the time courses of the BOLD-signals of those respective regions at rest. For the analyses, the time courses of the BOLD-signals at rest are filtered between 0.01-0.1 Hz to retain the low-frequency fluctuations. This is done for all regions-of-interest. Next, for every region-of-interest, correlation coefficients between these low-frequency fluctuations are calculated. These correlation coefficients are Fisher-z-transformed. 

**Figure 1:**
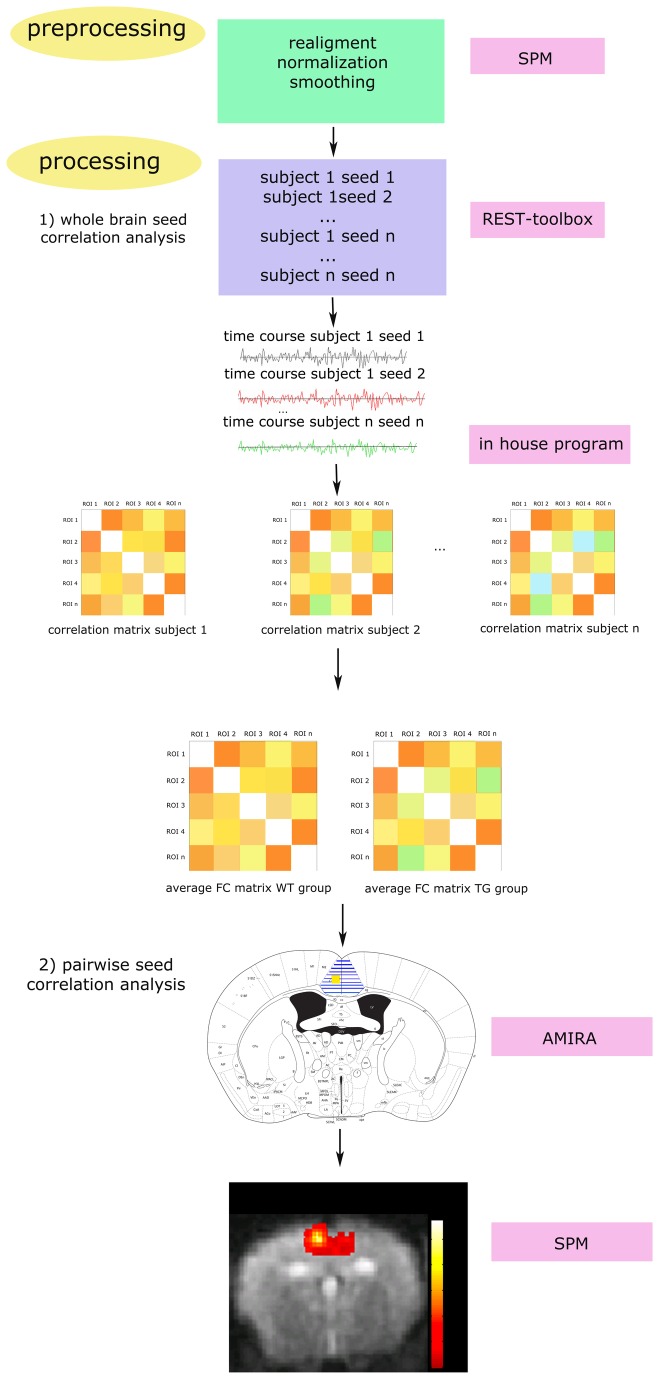
rsfMRI analysis. After the pre-processing steps, all rsfMRI data were analysed using the REST-toolbox. 1) Whole brain seed correlation analysis: The smoothed data and masks for every seed region were loaded and the filter was set between 0.01 and 0.1 Hz. Time courses of the low frequency fluctuations of the BOLD-signal were computed for each seed region from each subject. The functional correlation between the time courses of each seed region was calculated and the correlation coefficients were z-transformed, resulting in functional connectivity (FC) matrices for each subject. Then, the mean z-transformed FC matrices were calculated for the WT and TG groups. 2) Pairwise seed correlation analyses: Interhemispheric FC (hatched regions) was assessed between each seed region in the left hemisphere (yellow) and the corresponding region in the right hemisphere. Then, mean z-transformed FC maps of the WT and TG groups were computed in SPM.

The rsfMRI data were analysed by a whole brain seed correlation analysis and pairwise seed correlation analysis (Figure **1**). 

### Whole brain seed correlation analysis

Amyloid pathology is extensively present in the entire brain of 18 months old APP/PS1 mice [21], therefore seed regions were chosen in several cortical and subcortical regions (Figure **S1**): the left and right side of the retrosplenial cortex, somatosensory cortex, motor cortex, cingulate cortex, auditory cortex, visual cortex, hippocampus and thalamus using AMIRA software. 

The “RESTing state fMRI data analysis toolkit” (REST1.7, http://resting-fmri.sourceforge.net) was used to analyse the rsfMRI data. The smoothed data and a mask for each seed region was loaded into the software and the motion parameters resulting from the realignment (translation and rotation) were included as covariates to correct for possible movement that occurred during the scanning procedure. The filter was set between 0.01 and 0.1 Hz to retain the low-frequency fluctuations of the time course of the BOLD-signal that are of interest when performing resting state FC studies. The time courses were extracted for each seed region and correlation coefficients were calculated. These correlation coefficients were z-transformed using an in-house program developed in MATLAB (MATLAB R2013a, The MathWorks Inc. Natick, MA, USA) and represented in a correlation matrix. Mean z-transformed FC matrices were calculated for the WT and TG groups. 

### Pairwise seed correlation analysis

Pairwise seed analyses were performed to analyse some interhemispheric correlations in more detail (Figure **1**). Based on the whole brain seed correlation analysis three functional connections were selected for the pairwise seed analyses: The interhemispheric FC between left and right motor cortex, the interhemispheric FC between left and right somatosensory cortex and the interhemispheric FC between left and right hippocampus. The seed regions that we selected were therefore the left motor cortex, somatosensory cortex and hippocampus. Using the REST-toolbox, individual z-transformed FC maps were obtained for each seed region. These individual z-transformed FC-maps were loaded in SPM8 and mean statistical FC maps were computed for the WT and TG groups using a two-sided one sample T-test. These FC maps were masked to enable the assessment of interhemispheric FC e.g. for the FC map of the left hippocampus seed a mask was used containing the left and right hippocampus. The mean FC-maps are presented by overlaying them on the EPI-image.

### Statistical analysis

#### Whole brain seed correlation analysis

The statistical analyses for the correlation matrices were performed using MATLAB (MATLAB R2013a, The MathWorks Inc. Natick, MA). One sample T-tests were performed for the mean correlation matrices of the WT and TG groups to retain the significant correlations per group. The false discovery rate (FDR) correction was used to correct for multiple comparisons. A two sample T-test was used to determine which functional correlations are significantly different when comparing the WT and TG groups (p<0.05, uncorrected for multiple comparisons).

#### Pairwise seed correlation analysis

The statistical analysis of the interhemispheric FC maps was performed in SPM8. Mean statistical FC-maps were obtained for the TG and WT groups using a two-sided one sample T-test, where for each group interhemispheric FC is shown. These mean FC maps were family-wise-error (FWE) corrected for multiple comparisons (p<0.05), using a threshold of at least 10 voxels. Only voxels that remained significant after correction for multiple comparisons were interpreted as significant. Interhemispheric FC was compared across TG and WT groups for each seed region using a two-sample t-test with a FWE-correction for multiple comparisons (p < 0.05) and a threshold of 10 voxels. 

#### Histology

Immunofluorescence staining was performed to identify the pathology. The animals were sacrificed by cervical dislocation. Whole brains of WT (N=3) and TG (N=3) mice were formalin fixed and paraffin embedded and 5µm slices were sectioned using a Leica paraffin rotary microtome. The sections were then stained according to a previously optimised protocol [22], using the following antibodies: a guinea pig anti-synapsin antibody (Synaptic Systems, 106 004) in combination with a Cy3-labeled donkey anti-guinea pig secondary antibody (Jackson ImmunoResearch, 706-165-148) to assess the state of the synapses, a rabbit anti-Iba-1 (ionized calcium binding adaptor molecule 1, Wako, 019-19741) in combination with an AF555-labeled donkey anti-rabbit secondary antibody (Invitrogen, A-31572) to visualise microglia and a polyclonal rabbit anti-GFAP (glial fibrillary acid protein, Abcam, ab7779) in combination with an AF555-labeled donkey anti-rabbit secondary antibody (Invitrogen, A-31572) to visualise astrocytes. Sections were then stained using thioflavine-S (Sigma, T1892) to visualise amyloid plaques and counterstained using TOPRO-3 (Invitrogen, 642/661) to visualise the cell nuclei. Qualitative assessment of the immunofluorescence images was performed on a standard fluorescence microscope (Olympus BX51 fluorescence microscope) equipped with an Olympus DP71 digital camera.

## Results

The main focus of this work was to compare FC across TG and WT mice. For this purpose we analysed rsfMRI data using a whole-brain approach, i.e., a whole brain seed correlation analysis. We then used a pairwise seed analysis to examine three interhemispheric functional connections in more detail.

### Whole Brain Seed correlation Analysis

**Figure 2:**
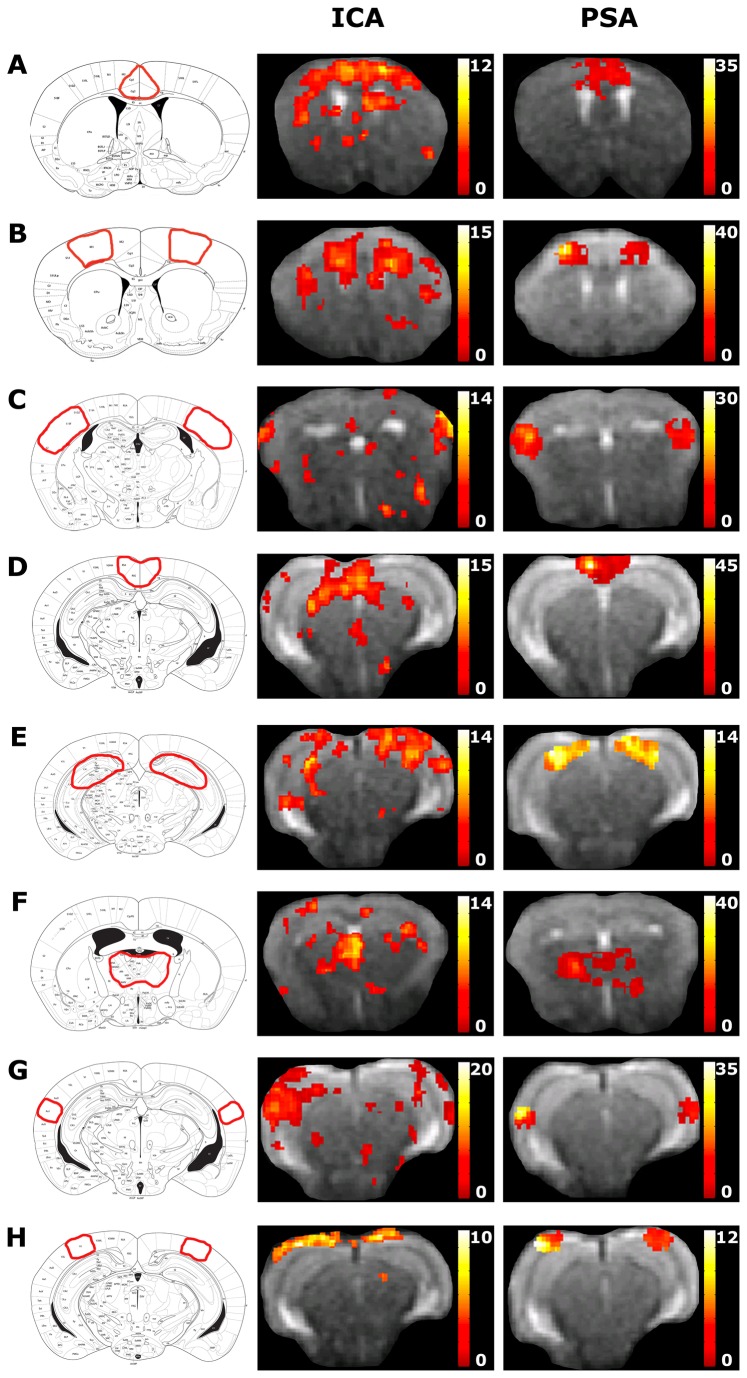
Results of the whole brain seed correlation analysis for the WT and TG groups. A) The FC matrices representing the mean correlation values between each seed region for the WT group and the TG group (FDR-corrected p < 0.05). The last figure of panel A shows the functional correlations that are significantly different between the WT and TG groups (p <0.05). The colour scale on the right represents the strength of the correlation. The functional correlation coefficients were calculated between the cingulate cortex (Cg), the motor cortex (MC), the somatosensory cortex (SC), the thalamus (T), the hippocampus (Hc), the temporal auditory cortex (AU), the visual cortex (VC) and the retrosplenial cortex (Rs) for the left (l) and the right (r) hemisphere. B) Grey lines represent correlation strength between seed regions (red dots). The thickness of the line is proportionate to the strength of the correlation: The thickest lines represent correlation coefficients > 0.35, the intermediate lines represent correlation coefficients 0.15–0.35, and the thinnest lines represent correlation coefficients < 0.15.

The mean correlation matrices and strengths of the correlations for the WT and TG groups are shown in Figure **2**. In general, FC was weaker for the TG group than for the WT group, across the entire brain. 

In some brain regions, including the motor cortex, interhemispheric FC appeared relatively well preserved in the TG group, whereas in other brain regions, including the somatosensory cortex and the hippocampus, interhemispheric FC was not well preserved in the TG group. We used a pairwise seed correlation to analyse these interhemispheric FCs in more detail.

### Pairwise seed correlation analysis

**Figure 3:**
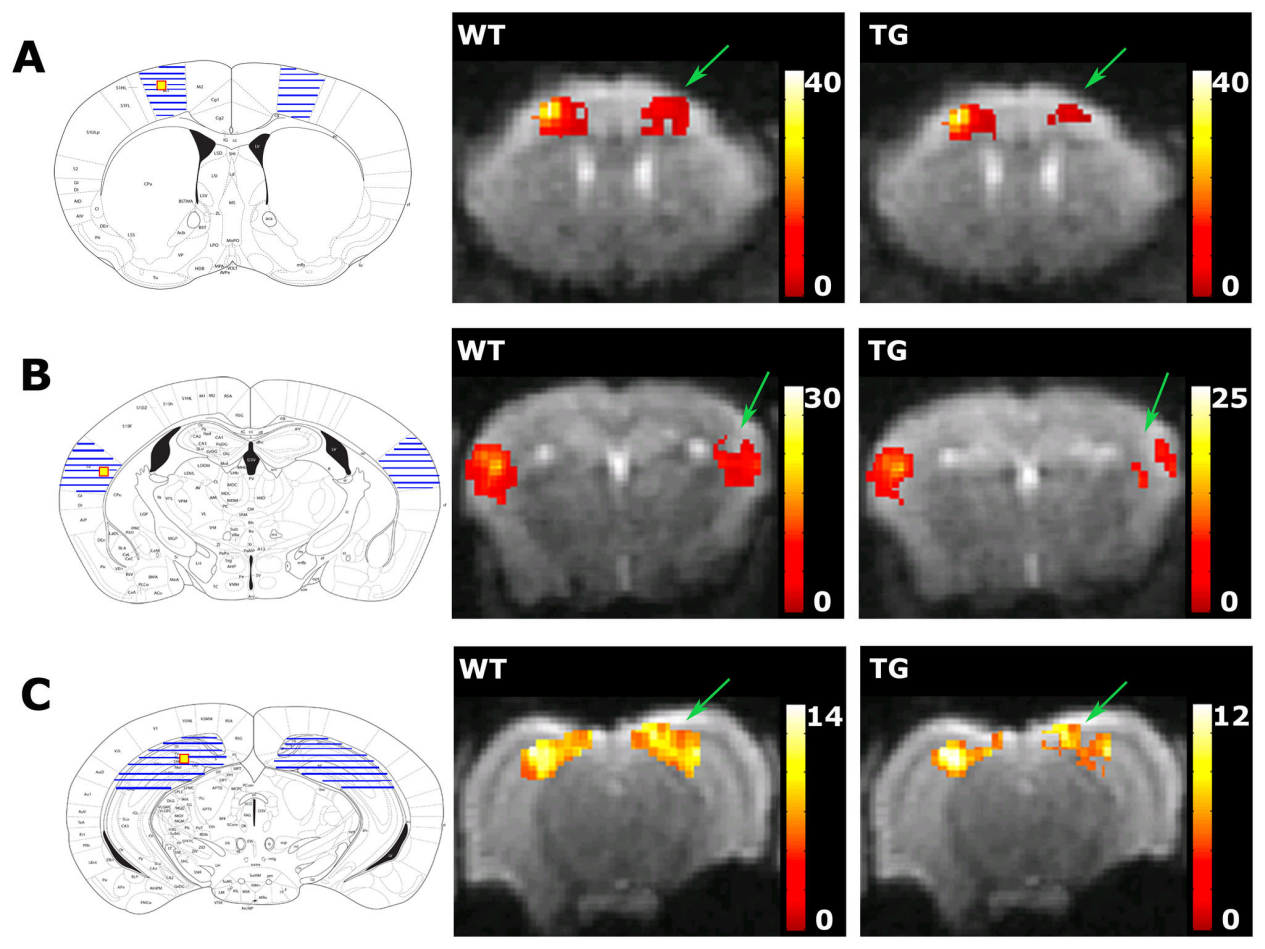
Results of the pairwise seed correlation analysis for the WT and TG groups. A = the motor cortex (MC), B = the somatosensory cortex (SC), C = the hippocampus (Hc). The left figure shows the location of the seed region (yellow) and the regions that were selected to assess interhemispheric FC (hatched) on the Franklin and Paxinos anatomical mouse brain atlas. The middle figure shows the mean statistical interhemispheric FC map of the WT group and the right figure shows the mean statistical FC map of the TG group. The mean FC maps for both groups are overlaid on an EPI image. The colour bar on the right represents the t-value, i.e., a measure of the strength of the functional correlation. The interhemispheric FC of the MC was similar in WT and TG groups. The interhemispheric FC of the SC and the Hc was lower in the TG group (FWE-corrected p < 0.05) than in the WT group (indicated by the green arrows).

The pairwise seed analysis indicated that although interhemispheric FC in the motor cortex was slightly altered in TG mice compared to WT mice, this did not reach statistical significance (Figure **3A**). Interhemispheric FC in the somatosensory cortex (FWE-corrected p = 0.036) and the hippocampus (FWE-corrected p = 0.009) was significantly lower in TG mice than in WT mice (Figure **3B and C**).

### Histology

**Figure 4:**
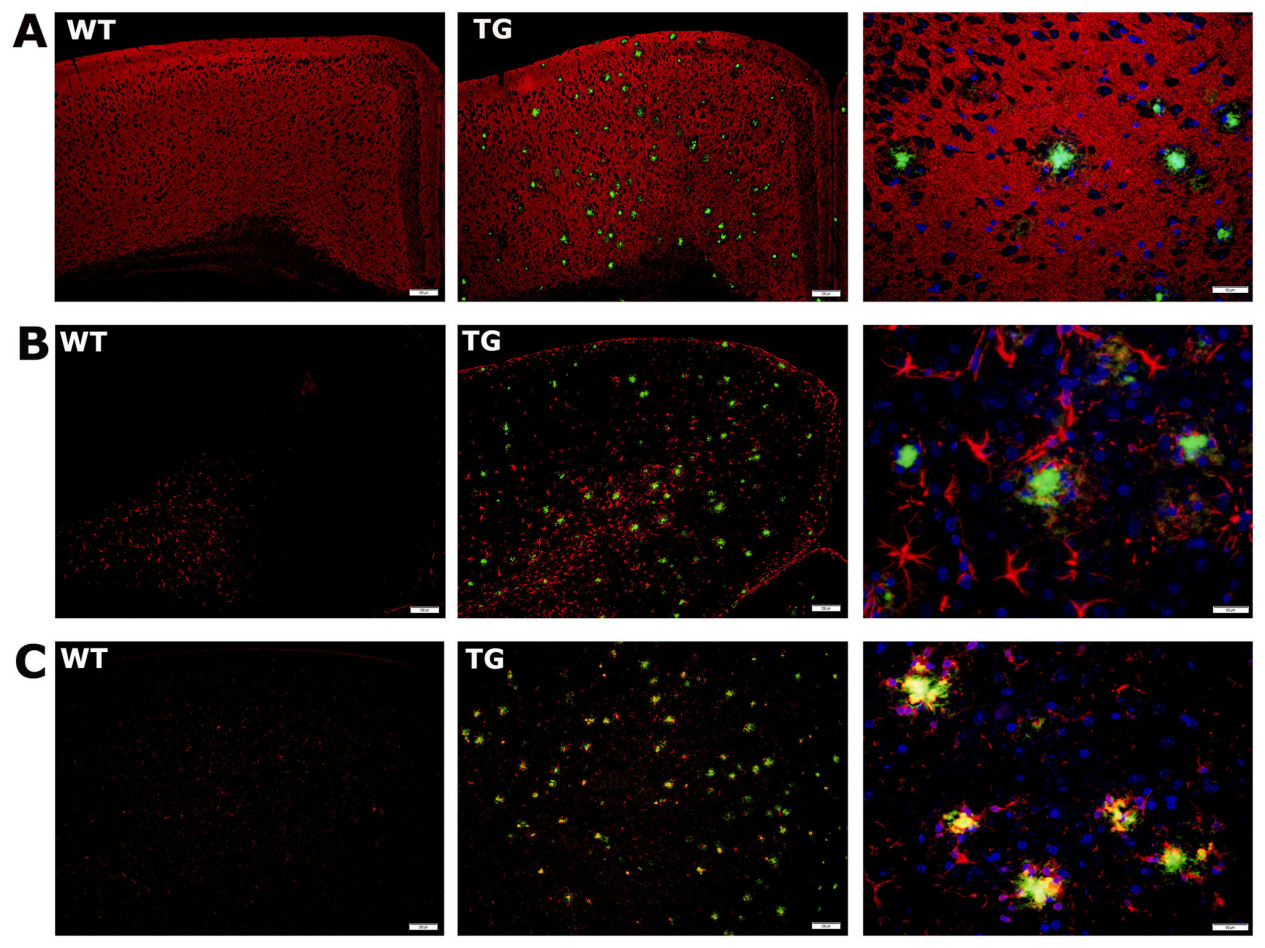
Results of immunofluorescence staining of the left sensory cortex for a representative WT and TG animal. A) The synapses (red) and amyloid plaques (green) for the WT group and TG group. Scale bar indicates 200 µm. The third column shows a close-up of an amyloid plaque and the surrounding synapses (scale bar indicates 50 µm). B) Astrocytes (red) and amyloid plaques (green) for the WT group and TG group. Scale bar indicates 200 µm. The third column shows a close-up of a plaque and the surrounding astrocytes (scale bar indicates 50 µm). C) Microglia (red) and amyloid plaques (green) for the WT group and TG group. Scale bar indicates 200 µm. The third column shows a close-up of a plaque and the surrounding microglia (scale bar indicates 50 µm), in which the cell nuclei are shown in blue.

The results of immunofluorescence staining of the cortex are shown for a representative WT animal and a representative TG animal in Figure **4**. Amyloid plaques were not detected in WT animals but were abundant in TG animals. Similarly, gliosis was clearly more abundant in TG animals than in WT animals. A close-up image of an amyloid plaque and the surrounding synapses shows the absence of synapses in the area around the plaque (Figure **4A**). Other close-up images show astrocytes residing in close vicinity of the amyloid plaques and engulfing them (Figure **4B**). Microglia were also observed close to the amyloid plaques and had a rounded shape, indicative of an activated state (Figure **4C**). This pathological trend was observed for all TG animals and for all cortical and non-cortical regions that were assessed. The images shown in Figure **4** are representative of the immunofluorescence analysis of all regions assessed in the current study.

## Discussion

We have evaluated FC in the brain of APP/PS1 mice, which is a mouse model for amyloidosis. We studied APP/PS1 mice at an age when amyloid pathology is extensively present throughout the entire brain and we used a whole brain seed correlation analysis to demonstrate a significant decrement in overall brain FC compared to WT mice. Next, we used a pairwise seed correlation analysis to focus on the motor cortex, an interhemispheric connection that was relatively preserved in TG mice according to the whole brain analysis, and the somatosensory cortex and hippocampus, which had interhemispheric connections that were diminished in the TG mice according to the whole brain analysis. The pairwise seed correlation analysis confirmed that interhemispheric FC in the motor cortex was preserved in TG mice, and that interhemispheric FC in the somatosensory cortex and hippocampus was diminished in TG mice. 

In our previous rsfMRI study [11] we used ICA to evaluate FC in the healthy mouse brain and showed that some components are bilateral (e.g. the motor cortex) whereas other components are only unilateral (e.g. the somatosensory cortex). In the current study, we have additionally performed an ICA analysis using the same protocol as described in our previous study by Jonckers et al. We have evaluated the presence of bilateral components in the wild-type group and have compared the results with an analysis performed using a pairwise seed correlation analysis. The results (Figure **S2**) show that, consistent with our previous ICA study, bilateral components could be distinguished for the motor cortex, the cingulate cortex, the retrosplenial cortex and the hippocampus. The components of the somatosensory cortex, the auditory cortex and the visual cortex showed some bilateral functional correlations, but, as in our previous ICA study, one side showed more dominance. The thalamus component, which was not discussed in our previous study, was also unilateral. The ICA results do not imply that interhemispheric FC is absent for some regions in the mouse brain; using a pairwise seed correlation analysis, bilateral FC could be observed for all the assessed regions (Figure **S2**). Furthermore, while the seed-based analysis did reveal group differences, statistical analyses of the ICA components did not show significant differences between the two groups. Discrepancies between the results of both techniques might be explained by differences in methodology [23] i.e. the requirements for data pre-processing are different for both techniques. Furthermore, the results of ICA strongly depend on a variety of factors including the number of pre-set components, selection of components that are considered to be neurologically relevant, the number of animals that are used, the type of anaesthesia etc. This is reflected by the results of a recent study [24], where both ICA and seed-based analyses revealed bilateral FC and FC networks in halothane anaesthetized mice; the major difference between both studies is the use of a different anaesthesia regime, combined with a different components pre-set and a higher number of animals. These are all factors that are important and interesting to investigate, but this is not within the scope of the current study. In this work we have used analysis techniques that do allow us to evaluate interhemispheric FC i.e. seed-based analyses.

APP_KM670/671NL_PS1_L166P_ mice are a model for amyloidosis, with amyloid plaque deposition in the neocortex starting at 6–8 weeks of age and amyloid plaque deposition in the hippocampus and the striatum starting at 8–12 weeks of age. By 19 months of age the entire brain is covered with amyloid plaques [21]. Besides amyloid plaque deposition, these TG mice also show microgliosis, astrocytosis [25] and hyperphosphorylated tau-structures that do not develop into full tau-pathology [21]. Loss of dendritic spines [26] and modest local neuron loss in the dentate gyrus have also been reported but global neuron loss does not occur in these mice [27]. Synaptic transmission defects were reported to occur in cultured hippocampal neurons from these TG mice [28] and synaptic plasticity was disrupted in the hippocampus of 8 months old TG mice [29]. Thus, according to the literature, amyloidosis and the consequential gliosis and synaptic defects may be the main pathological events underlying FC alterations in APP_KM670/671NL_PS1_L166P_ TG mice.

Our immunofluorescence histological analyses showed an abundance of amyloid plaques in all brain regions in the TG mice, whereas no plaques were observed in the WT mice. We observed an absence of the presynaptic marker synapsin in the area surrounding the plaques. This could mean that the synapses had been lost due to neurodegeneration, or that the synapses had been displaced due to the formation of amyloid plaques. Microgliosis and astrocytosis were also more extensive in the TG mice, and surrounded the amyloid plaques. Although the histological analyses were not quantitative, these data suggest a link between FC deficits and amyloid pathology and the consequential inflammatory responses and synaptic deficits. 

In AD patients altered FC was demonstrated within and between several resting state networks. These resting state networks include the default mode network, which includes the posterior cingulate cortex and the medial thalamus [30]. The default-mode network shows strong functional connections with the hippocampus in the healthy brain [31], and this is impaired in AD patients [31–34]. Other resting state networks that are impaired in AD include the salience network, which includes the anterior cingulate cortex and the sensorymotor network, which consists of the primary visual and auditory cortices [30]. The link between FC and amyloid pathology has been suggested by studies that have demonstrated altered FC in the brain of asymptomatic subjects that harbour amyloid plaques [18,35]. However, a recent study that combined rsfMRI and positron emission tomography showed no correlation between FC deficits and amyloid plaques [36]. These findings suggest that other AD-related pathological alterations might lead to a cascade of events that eventually cause FC deficits; for example, the soluble form of amyloid is known to exert toxic effects at the level of the synapses [17] which could lead to synaptic deficits and eventually FC deficits. 

The next step in this field is to perform longitudinal studies to correlate rsfMRI results with specific stages of amyloid pathology. Such studies may also be helpful in assessing why no FC deficits were observed in the motor cortex where, according to the histology results, pathology was present. Bero and colleagues [13] demonstrated that regional amyloid accumulation was related to the age-dependent reduction of bilateral functional correlations in another APP/PS1 mouse model [37]. In their study, the visual cortex showed less amyloid pathology than other brain regions, and there was no significant age-related decline in FC in this region. Although the studies are not fully comparable due to the use of different techniques, setup, and mouse models, among other factors, the same rationale could be true for the current study, i.e., it is possible that amyloid pathology was present in the motor cortex, but present to a lesser extent than in other brain regions, thus explaining why FC was preserved in the motor cortex. Nonetheless, our study demonstrated that FC was affected in several brain regions of APP/PS1 mice, at an age when amyloid pathology was present in the entire brain. 

## Conclusion

In the current study, we used rsfMRI to show altered FC in several brain regions in a mouse model of amyloidosis. Further studies that longitudinally assess FC at different stages of amyloid pathology will provide more insight into the exact relation between FC and amyloidosis. 

## Supporting Information

Figure S1 **Location of the seed regions.** The location of the seed regions is illustrated on the Franklin and Paxinos anatomical mouse brain atlas. Seed regions were placed in the motor cortex (khaki; interaural 4.66; bregma 0.86), the cingulate cortex (blue; interaural 3.10; bregma -0.70), the thalamus (purple; interaural 3.10; bregma -0.70), the somatosensory cortex (brown; interaural 2.58; bregma -1.22), the retrosplenial cortex (green; interaural 1.34; bregma -2.46), the auditory cortex (red; interaural 1.34; bregma -2.46), the visual cortex (yellow; interaural 1.10; bregma -2.70 ) and the hippocampus (fuchsia; interaural 1.10; bregma -2.70 ) in the left and right hemisphere.
(TIF)

Figure S2 **Comparison of bilateral FC using ICA and a pairwise seed correlation analysis.** The regions of which bilateral FC was assessed are illustrated on the Franklin and Paxinos anatomical mouse brain atlas (left). Bilateral FC was assessed using ICA (middle) and pairwise seed correlation analysis (PSA) (right) for the cingulate cortex (A), motor cortex (B), somatosensory cortex (C), retrosplenial cortex (D), hippocampus (E), thalamus (F), auditory cortex (G) and visual cortex (H). The colour bar indicates the t-value and is a measure for the strength of the functional correlation. ICA was performed as described in our previous ICA study [11]. The pairwise seed correlation analysis was performed as described in the material and methods section, but additional seed regions were included.
(TIF)
