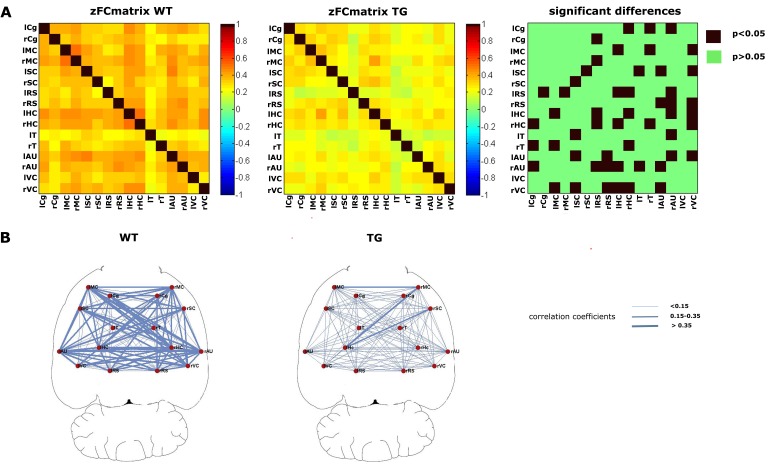# Correction: Resting State fMRI Reveals Diminished Functional Connectivity in a Mouse Model of Amyloidosis

**DOI:** 10.1371/annotation/5bfdca66-ef38-403a-a370-9c273d878e4a

**Published:** 2014-01-10

**Authors:** Disha Shah, Elisabeth Jonckers, Jelle Praet, Greetje Vanhoutte, Rafael Delgado y Palacios, Christian Bigot, Dany V. D’Souza, Marleen Verhoye, Annemie Van der Linden

Figure 2, "Results of the whole brain seed correlation analysis for the WT and TG groups," was mistakenly replaced by a duplicate of Figure S2. The Figure 2 legend is correct. Please see the correct Figure 2 here: 

**Figure:**