# Supplementary figures and images for: Resting State fMRI Reveals Diminished Functional Connectivity in a Mouse Model of Amyloidosis
Source: PLoS One. 2013 Dec 17;8(12):e84241. doi: 10.1371/journal.pone.0084241 (PMC3866274; doi:10.1371/journal.pone.0084241)

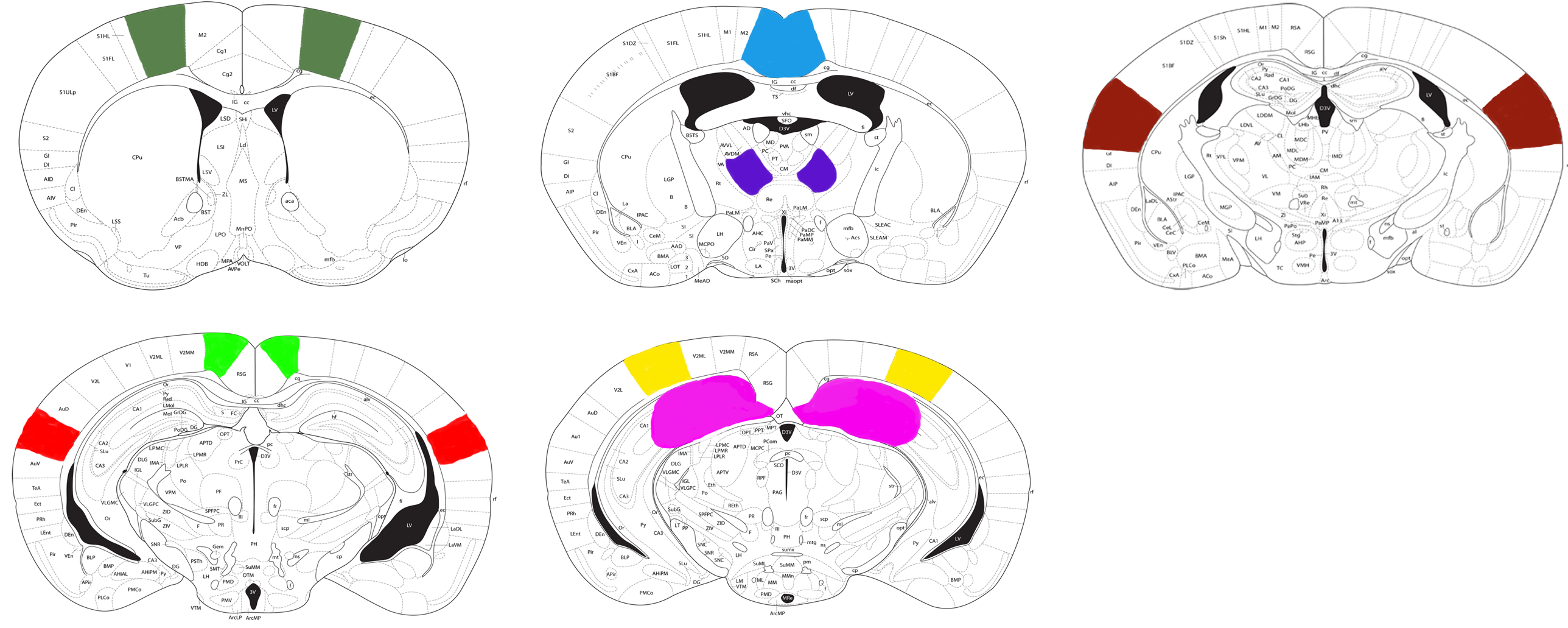

Supplement: Figure S1 — Location of the seed regions. The location of the seed regions is illustrated on the Franklin and Paxinos anatomical mouse brain atlas. Seed regions were placed in the motor cortex (khaki; interaural 4.66; bregma 0.86), the cingulate cortex (blue; interaural 3.10; bregma -0.70), the thalamus (purple; interaural 3.10; bregma -0.70), the somatosensory cortex (brown; interaural 2.58; bregma -1.22), the retrosplenial cortex (green; interaural 1.34; bregma -2.46), the auditory cortex (red; interaural 1.34; bregma -2.46), the visual cortex (yellow; interaural 1.10; bregma -2.70 ) and the hippocampus (fuchsia; interaural 1.10; bregma -2.70 ) in the left and right hemisphere. (TIF) [file pone.0084241.s001.tif]

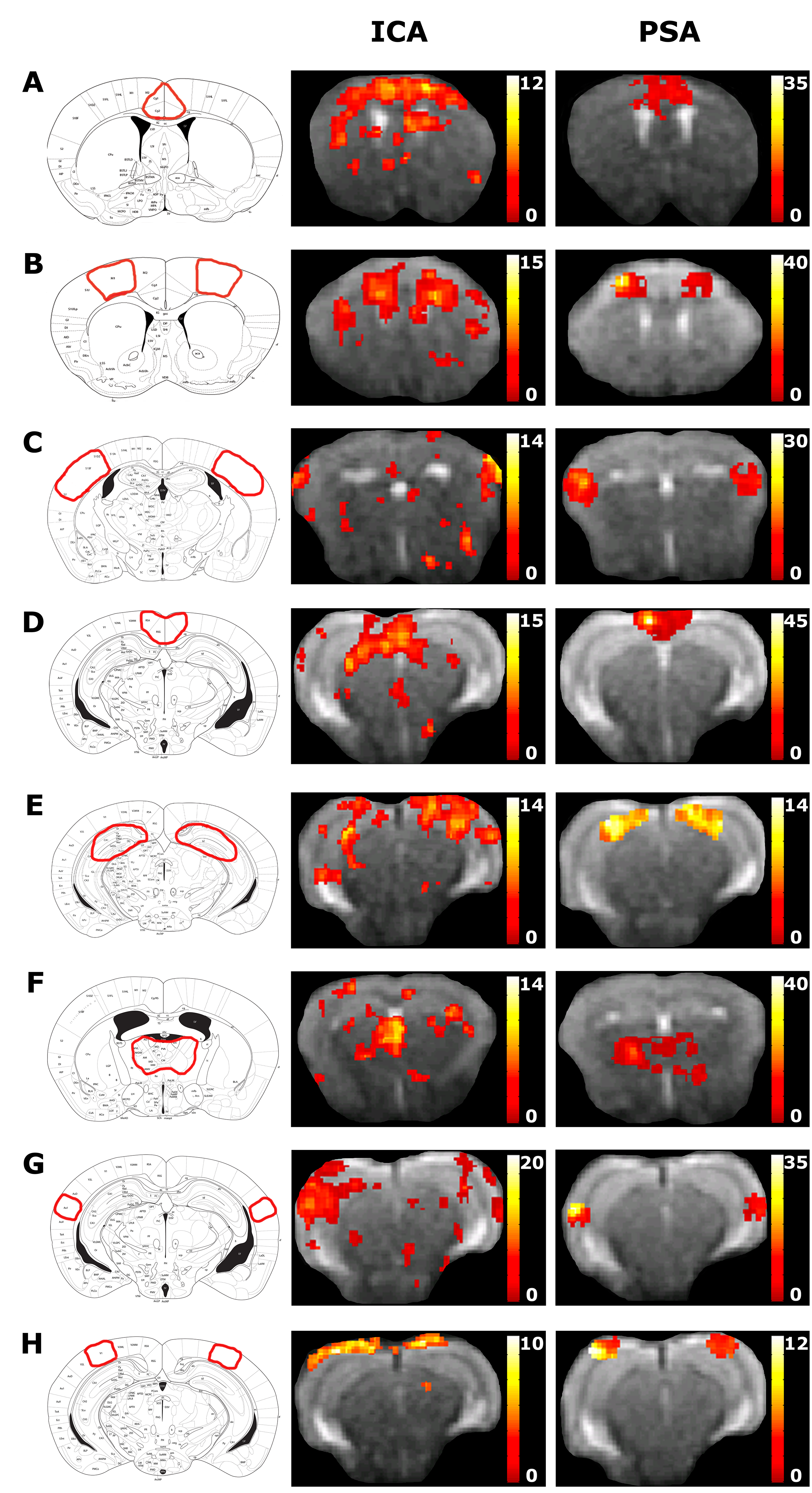

Supplement: Figure S2 — Comparison of bilateral FC using ICA and a pairwise seed correlation analysis. The regions of which bilateral FC was assessed are illustrated on the Franklin and Paxinos anatomical mouse brain atlas (left). Bilateral FC was assessed using ICA (middle) and pairwise seed correlation analysis (PSA) (right) for the cingulate cortex (A), motor cortex (B), somatosensory cortex (C), retrosplenial cortex (D), hippocampus (E), thalamus (F), auditory cortex (G) and visual cortex (H). The colour bar indicates the t-value and is a measure for the strength of the functional correlation. ICA was performed as described in our previous ICA study [11]. The pairwise seed correlation analysis was performed as described in the material and methods section, but additional seed regions were included. (TIF) [file pone.0084241.s002.tif]
